# Reply to Vanderberg, “Further Mechanisms and Locations in Which Antisporozoite Antibodies Neutralize Malaria Sporozoites”

**DOI:** 10.1128/mBio.02108-19

**Published:** 2019-09-10

**Authors:** Photini Sinnis, Fidel Zavala

**Affiliations:** a Johns Hopkins School of Public Health, Baltimore, Maryland, USA; University of California Los Angeles

## REPLY

We would like to respond to the letter from Dr. Vanderberg in which he suggests that an additional mechanism by which circumsporozoite protein (CSP) antibodies act at the inoculation site is by impacting the inoculation of sporozoites by infected mosquitoes. In their 2009 paper (1), they find that high concentrations of CSP antibodies in the rodent host lead to fewer sporozoites inoculated by infected mosquitoes due to the agglutination of sporozoites as they are inoculated. While we do not doubt the validity of these results, the experimental conditions we used differ significantly, and therefore, those findings may not be relevant to our conclusions (2). Specifically, we used significantly lower amounts of CSP antibodies, resulting in concentrations more in line with levels observed in RTS,S-vaccinated individuals (3). In the Vanderberg study, passive immunization was performed with 320 μg of antibody *in vivo*, and *in vitro* studies demonstrating sporozoite agglutination used 1 mg/ml of antibody. In our studies, in which we compared sporozoite infectivity in immunized and naive mice after challenge with mosquito- and intravenous-inoculated sporozoites, passive immunizations were performed with 12 to 50 μg of antibody. At these doses, serum antibody levels would be 6 to 25 times lower than those used in the Vanderberg study. Importantly, at these antibody concentrations, we did not observe any agglutination of sporozoites when they were inoculated by using a needle and observed by intravital microscopy. Thus, while we do not doubt the previous findings of Vanderberg et al. (1), we do not find them relevant to our work.

## References

[1] Kebaier C, Voza T, Vanderberg JP 2009 Kinetics of mosquito-injected *Plasmodium* sporozoites in mice: fewer sporozoites are injected into sporozoite-immunized mice. PLoS Pathog 5:e1000399. doi:10.1371/journal.ppat.1000399. 19390607 PMC2667259

[2] Flores-Garcia Y, Nasir G, Hopp CS, Munoz C, Balaban AE, Zavala F, Sinnis P 2018 Antibody-mediated protection against *Plasmodium* sporozoites begins at the dermal inoculation site. mBio 9:e02194-18. doi:10.1128/mBio.02194-18. 30459199 PMC6247089

[3] White MT, Verity R, Griffin JT, Asante KP, Owusu-Agyei S, Greenwood B, Drakeley C, Gesase S, Lusingu J, Ansong D, Adjei S, Agbenyega T, Ogutu B, Otieno L, Otieno W, Agnandji ST, Lell B, Kremsner P, Hoffman I, Martinson F, Kamthunzu P, Tinto H, Valea I, Sorgho H, Oneko M, Otieno K, Hamel MJ, Salim N, Mtoro A, Abdulla S, Aide P, Sacarlal J, Aponte JJ, Njuguna P, Marsh K, Bejon P, Riley EM, Ghani AC 2015 Immunogenicity of the RTS,S/AS01 malaria vaccine and implications for duration of vaccine efficacy: secondary analysis of data from a phase 3 randomised controlled trial. Lancet Infect Dis 15:1450–1458. doi:10.1016/S1473-3099(15)00239-X. 26342424 PMC4655306

